# The Nexus of Service Quality, Customer Experience, and Customer Commitment: The Neglected Mediating Role of Corporate Image

**DOI:** 10.3389/fpsyg.2022.917284

**Published:** 2022-06-30

**Authors:** Yang Yingfei, Zhang Mengze, Bae Ki-Hyung

**Affiliations:** ^1^College of International Economics and Trade, Ningbo University of Finance and Economics, Ningbo, China; ^2^Department of Economics, Sejong University, Seoul, South Korea

**Keywords:** customer experience, customer commitment, corporate image, service quality, logistics

## Abstract

Quality of service is a major determinant of customer commitment to the organization. Therefore, it is important to understand the importance of service quality for the corporate image as well. In this study, the predicting roles of quality of service and customer experience have been unveiled in customer commitment through the mediating effect of corporate image. The population frame used in this study is the customers of logistic services providers in China. Total data from the 366 customers have been used to analyze the hypotheses formulated. The sample has been selected using convenience sampling and the software used for data analysis is Smart-PLS. The analytical technique used is partial least square structural equation modeling. Results of the study show that service quality and customer experience have a significant role in the customer commitment to the suppliers. In addition, it has also been found that service quality and customer experience have a major contribution to building the corporate image of the services suppliers. Further, corporate image played a significant mediating role in the relationship between service quality and customer commitment. The study has theoretically contributed to the body of literature by finding the importance of service quality for predicting customer commitment to the suppliers.

## Introduction

Price, quality, and functionality are all important product/service features, and their implications on customer behavior have already been studied extensively by scholars and practitioners. Conversely, in the 20th century, there was a significant shift out from a strict transactional to a more relational perspective. This shift toward commitment emphasizes on the value of creating and sustaining a greater commitment of customers instead of only transactions done by them (Coviello et al., 2002). More recently, a new trend has emerged, with a greater emphasis on customer experience, such as a crucial organizational essential (Klaus and Maklan, 2013). Commitment is an important factor in establishing and managing long-term meaningful relationships, according to mainstream consumer research (Alamgir and Uddin, 2017).

Likewise, in customer-based organizational management, commitment is seen as a necessary prerequisite for achieving desirable outputs like the confirmation process, prospective intents, and revenue generation. It has also been evident that there is a rising prevalence of research in marketing that examines brand commitment (Rucker et al., 2014). In the domain of organizational management, this study utilizes a suitable theoretical framework consisting of the affective, calculative, and normative dimensions of customer commitment. This research is consistent with the prior study which classifies the principle of commitment as a multidimensional concept (Eisingerich and Rubera, 2010).

In a variety of areas, including marketing the framework is commonly used for empirical study. The three aspects of commitment are built on well-defined conceptions that encompass both subjective and attitudinal (calculative and prescriptive) elements of customer behavior (Allen and Meyer, 1990). It is assumed that customer commitment could be achieved through service quality and customer experience (CE). Management of the customer experience, which is simply described as producing a positive customer experience is being heralded as the new frontier in which businesses will compete for a share of the market. According to Keiningham et al. (2017), 89% of businesses intended to operate largely based on the experiences of customers. Furthermore, management of customer experience has recently arisen as a unique specialty in response to the constantly increasing demand for knowledge in the industry by organizations (Fatma, 2014). Despite this focus, agreement among managers and academics about what constitutes customer experience, how it is measured, and how it varies from contextual factors is developing, but it has yet to be studied. Therefore, there is no broad agreement on which parts of CE require evaluation and analytics. There is also no evidence available on the relationship between CE and other more recognized business frameworks (Gao et al., 2021). Authors feel that comprehending how CE fits into the comprehensive business literature requires research in more established marketing structures (Keiningham et al., 2017).

The importance of CE in the marketing of brands has gained a lot of theoretical and empirical backing. Nevertheless, some disagreement prevails over its ability to generate brand loyalty (Lemon and Verhoef, 2016). In a research conducted by Francisco-Maffezzolli et al. (2014), it was identified that there was an indirect association of CE with the commitment of the customers. Moreover, it was also assessed that the direct effects of CE created a huge ambiguity toward customer commitment (Ramaseshan and Stein, 2014), which exposed a significant gap in the research for the direct association of both the variables. There has been a need to explore the direct relationship of CE with customer commitment. It is to evaluate the impact of customers' experience with the brand and their commitment to the brand. It will contribute to future studies if this association comes out significant. The companies will start focusing on this aspect for providing a better experience to the customers. The market is fast evolving, and we are now living in a period of a thriving multinational service economy that prioritizes service quality (Hsu et al., 2006).

Recognizing what customers expect from a service can help businesses allocate resources and make adjustments depending on customer needs. As a result, a thorough grasp of what a client considers to be greater service became a critical concern and demand for every business or company's process (Chien and Chi, 2019). According to a related study, improving service quality became one of the most important management strategies for increasing customer commitment and engagement (Su and Teng, 2018). It is one of the most important variables impacting a company's performance (Ismail et al., 2021). Previously, service quality has not been explored as having impact on customer commitment; rather, it was tested along with customer commitment for evaluating customer loyalty in many cases (Izogo, 2017).

This posed a huge gap that both are the different aspects of organizational management and should be tested in a way to evaluate the role of service quality toward customer commitment. It has been observed that service quality was associated with customer satisfaction, loyalty, and perceived value in past (Özkan et al., 2020). According to Reichheld (1993), the main objective of the business management teams is to determine whether the services supplied to the customers have any link to the demands of the customers or not. This kind of service could have provided a competitive edge to the service providers in the marketplace due to the loyalty factor. The commitment of the customers could be utilized as an assessment approach through which the reactions of the customers could be assessed as an aggregate approach for the services and products (Roy et al., 2022). In a consumer market, offering high-quality service which results in delighted consumers is the key to long-term competitive advantage. The service quality provided all along the supply chain may aid in the development of loyal customers, which would lead to corporate success. Service quality is linked to corporate success, budget savings, customer happiness, consumer loyalty, and profits (Nguyen et al., 2018). Service quality is an evaluative phenomenon of bi-directional exchanges, involving service users and service providers at the dyadic level (Prakash, 2019). Hence, it is assumed that providing good quality services to the customers could influence their commitment levels. Moreover, with the influence of service quality, the thing which may boost the customer commitment is the corporate image as suggested by Chien and Chi (2019).

Previously, the corporate image has been explored as a mediator between service quality and customer satisfaction (Chien and Chi, 2019), which suggested its further utilization as a mediator, leaving a gap in marketing research for service quality provision. This kind of mediating role of corporate image helps provide the best services to the customers. If companies possess a good corporate image, then it helps the customers to make decisions about the purchase of their products. Therefore, utilizing corporate image as a mediator helps shape the service-customer relationships. In a scholarly study, the reputation of the company or image, customer happiness, and firm performance are all very significant (Chien and Chi, 2019). Scholars defined image as a general characteristic of a firm that makes reference to which a firm is seen as excellent or negative (Alamgir and Uddin, 2017). To be more specific, corporate image relates to the public's perceptions of a corporation (Alamgir and Uddin, 2017). Kim et al. (2020) argue that image or repute is significant because it shows how a business is compared with its competitors in terms of stakeholder perceptions of the company's willingness to operate in a given way.

The corporate image may influence a company's capability to increase pricing without losing customers (Kim et al., 2020). Corporate image has been associated with a lot of variables studied in past such as loyalty, service quality, customer satisfaction, and so on. Majorly, it has been studied as a mediator between service quality and customer satisfaction (Chien and Chi, 2019). It was also observed that corporate image along with corporate reputation was more concerned with organizations associated with the service sector (Özkan et al., 2020). Developing a stronger corporate image with such a solid organizational reputation is especially crucial for service firms in this regard. It is thought to be considerably aiding in establishing client commitment (Yasin, 2021). Therefore, the use of corporate image as a mediator between service quality and customer commitment is useful.

Customer commitment is typically improved by positive opinions of how a firm operates, and financial service organizations are familiar with building/maintaining a positive image of the company and maintaining a solid corporate reputation will provide a long-term competitive edge. As a result, a good company image and corporate reputation are valuable assets for a service business, because clients have many options (Özkan et al., 2020). Keeping in view the significance of corporate image in developing customer commitment in China, this research utilized corporate image as a mediator. A corporate image is a powerful tool of CSR in organizational processes. It helps in developing a bond between customers and the enterprise. This research helps practice corporate social responsibilities in enterprises. Therefore, it will add to the literature of CSR-based firms. This research is based on some research questions which are given as follows:

RQ1. How service quality and customer experience can play a role toward customers' commitment?
RQ2. How corporate image helps in mediating the relationships of service quality, customer experience, and customer commitment?

To answer these questions, this study tries to find out the possible relationships between service quality, customer experience, and customer commitment. This research also figured out the mediating role of corporate image in between service quality, customer experience, and customer commitment.

## Theoretical Support

The expectation disconfirmation theory (EDT) by Oliver and DeSarbo (1988) is a basic theory used by marketing and consumer behavior experts. EDT theory is defined as a unit of set expectations in relation to confirmation or disconfirmation (Oliver and DeSarbo, 1988). Expectations are a collection of beliefs that a consumer has about a service or product and disconfirmation is the contrast between pre-consumption perception and post-consumption reality. This disparity may have both beneficial and bad implications. Positive variance obtained through analysis indicates that the experience of the customers after consuming the products is better than the expectations which they had before consumption. Similarly, the negative variance obtained through analysis indicates that the experience after the consumption of products is not as good as the expectations were before the consumption (Yi et al., 2021).

Negative disconfirmation is discontent with a specific product or service; positive disconfirmation is contentment with products and services provided by the brands (Yi et al., 2021). Customer satisfaction leads to customer commitment to the product and the brand (Sigit Parawansa, 2018). Therefore, this theory has a significant contribution to customer commitment. Consumer behavior and marketing, human capital, recreational behavior, medical, sociology, service quality, brand management, and administration are just a few of the areas where the theory is applied (Uzir et al., 2021). Hence, we inferred that assessing the impact of service quality on customer commitment could be based on principles of EDT.

Customers expect an advantage or efficacy from a service or product that exceeds their expectations, according to EDT. The comparison indicates if clients are satisfied or dissatisfied with the service, then it leads to their commitment to the service brands. This commitment is determined by the level of service offered by the organization when delivering purchased products to customers, as well as how they view the company's service and image (Uzir et al., 2021). This research also draws support from the theory of cognitive psychology (Folkes, 1988). Based on this theory, it can be drawn that corporate image can filter the psychological dimension of customers toward buying the services and products. It may have a facilitating role in defining the commitment of customers to the company's services and products. Drawing on relationship marketing theory (Berry, 1995), this research seeks to investigate the possible relationships between service quality, customer experience, corporate image, and customer commitment. Since the 1970's, relationship marketing has been offered as a theory. It refers to the process of establishing, developing, and managing long-term, mutually beneficial connections between two entities. According to the theory, relationship marketing allows a company to gain a deeper understanding of individual customers' needs, enhancing its ability to meet customer aspirations with greater success (Saglam and El Montaser, 2021). Relationship management is no longer about gaining a greater portion of the customer's money, but instead about gaining a bigger share of the customer's mind, thoughts, and personal wealth. Customer commitment is the goal of relationship marketing, which may lead to recurrent purchases by loyal customers (Roberts-Lombard, 2020).

Long-term interactions among partners can be professionally handled through an engaged approach based on relationship marketing principles. It will also result in a better knowledge of their different needs and desires, lowering the chances of a failed relationship. As a result, relationship marketing seeks to gain satisfied customers who are willing to commit to a long-term connection with the company (van Tonder and Petzer, 2018). This research got support from these theories and tried to find out the possible relationships between service quality, customer experience, mediating corporate image, and customer commitment. These theories have a strong backing for customers' engagement and satisfaction which ultimately leads to improved and enhanced customer commitment to the services and products of the organizations. These relationships usually occur as an exchange between the customers and organizations.

### Service Quality, Corporate Image, and Customer Commitment

Service quality was described by Gronroos (1988) as the consequence of an assessment procedure in which consumers evaluate their aspirations with the services they consider to have ended up getting. It is defined as the level of quality of services and products given to consumers, as well as their level of satisfaction toward services. It is the product of the interplay between customers' conceptions of expected and perceived services, as well as the workable causal relationships such as technical ability and features of the product and image (Uzir et al., 2021). Service quality has been identified as a critical and focal aspect in customer company organizations by Omar et al. (2021).

Service quality (SQ) is a model and is referred to as SERVQUAL in different literature. This model is based on five dimensions such as assurance, empathy, tangibility, reliability, and responsiveness (Uzir et al., 2021). This model is utilized for measuring and capturing service quality for customers. Tangibility refers to the appearance of the service with reference to its surroundings which makes sure the existence of the service. Empathy refers to the attention given to the customers passionately or individually. Dependability and consistency of the services are referred to as reliability. The fourth dimension of SQ is responsiveness which refers to as the provision of the services in light of willingness to serve the customers. The last dimension is about assurance which deals with confidence and trust elements of service quality offered to customers (Kim, 2021).

According to SERVQUAL, service quality has a positive and important influence on customer satisfaction and commitment in a range of circumstances, such as on home service, hospitality business owners in Southeast Asia, the life insurance companies in Malaysia, the healthcare sector in India, eatery enterprises in Korea, and food retail in Chile, as well as the financial sector globally (Uzir et al., 2021). The SQ model, which has been used in a variety of service organizations to examine the influence of client satisfaction and other aspects of service quality, focuses on distinct dimensions of service quality. The numerous prominent characteristics of a service business have also been demonstrated to be the most promising aspects of consumer commitment (Yi and Nataraajan, 2018).

Many of the past studies have also focused on evaluating the impact of service quality on customer satisfaction which leads to customer commitment in the context of logistics and delivery of digital purchases (Buldeo Rai et al., 2019). Out of the two elements of corporate image, the first aspect is in terms of function and the second component is in terms of emotion. The features of an element are observable and may be measured. While the emotional principle has intangible characteristics associated with psychological characteristics such as feelings, attitude, and perception of the company, the psychological element has particular characteristics which are associated with psychological characteristics like sentiments, mindset, and image of the service (Chattananon et al., 2007).

Management literature proposed some well-known as well as similar brand image or corporate image models that identify the factors that influence a positive corporate image (Chattananon et al., 2007). Some of the scholars have also looked into the relationships between service quality and customer commitment and found significant associations (Chenet et al., 2010). This study found an association between service quality and different parameters of customer-related attributes which ultimately affected the customers' commitment to the services. Similarly, a few researchers found an association between service quality and corporate image and in this scenario, service quality showed a significant association with the corporate image (Özkan et al., 2020). A corporate image is a bridging tool between firms and customers. Customers are more oriented toward the firms which practice CSR in the processes. Therefore, based on this literature support, we proposed the following hypothesis.

***H***_**1**_ *: Service quality has a strong association with customer commitment*
***H***_**3**_ *: Service quality has a possible association with corporate image*

### Customer Experience, Corporate Image, and Customer Commitment

What people desire are not objects but gratifying experiences (Abbott, 1956). Historians expanded on this perspective in the 1980's by addressing the experience dimensions of services, which go beyond intellectual or cognitive events (Holbrook and Hirschman, 1982). The customer experience (CE) refers to any services and products exchange and hence encompasses the consumer's whole shopping experience. This includes a customer's intellectual, emotive, behavioral, and interpersonal reactions to brand-related activities. Numerous CE interpretations can be found in the literature, indicating a discrepancy on how to define it (Lemon and Verhoef, 2016). CE is a multifaceted concept that focuses on a customer's cognitive, emotional, behavioral, perceptual, and interpersonal reactions to a company's products throughout the entire buying process (Lemon and Verhoef, 2016).

The following remarks are based on the evaluated CE definitions. Most scholars regard CE as a very subjective, personal idea that varies depending on the interactions that make up the customer's whole buying process. Unlike engagement which represents a consumer's interest in a given brand contact, CE encompasses the whole purchasing experience of the customer (Hollebeek and Rather, 2019). CE is also commonly thought of as a multi-dimensional phenomenon that indicates a consumer's reaction to a specific product stimulus. CE is usually understood to have intellectual, emotive, behavioral, sensory, and social aspects; however, there is some controversy about its multiplicity. It seems to be the focused experiential topic which can vary from macro-level to micro-level items. Although CE may span the full customer experience, sub-experiences could concentrate on certain touch points during the purchase process (Khan et al., 2020).

The term commitment refers to an explicit or implicit promise by trading partners to maintain their connection indefinitely. According to Moorman et al. (1992), commitment is defined as a customer's desire to sustain a valuable relationship by exerting full effort in doing so. As a result, commitment implies the consumer's willingness to make concessions to ensure long interpersonal advantages, as well as the person's attachment to the brand. This kind of attachment with the brand makes him forgiving for the concerns attached to the brand. It also results in less influence by the competitive nature of the brands. Whereas, the meaning of customer commitment is up for discussion, authors find certain similarities in most approaches. Commitment refers to a person's natural desire to stay with their exchange relationship (Khan et al., 2020).

Second, client commitment is frequently regarded as a multi-faceted notion. Authors concentrate on the analytical and emotive commitments of clients, which fulfill their emotional and cognitive needs, accordingly. Normative commitment, on the other hand, focuses on concerns that customers believe they should do (Khan et al., 2020). Going through these definitions of customer commitment and customer experiences, it was assumed that customer experiences might have a direct relationship which is a dimension of the customer purchase journey. Moreover, keeping in view the functionality and significance of the corporate image, it was assumed that customer experience could affect both, corporate image and customer commitment. Therefore, the authors devise the following hypotheses:

***H***_**2**_ *: Customer experience has an impact on customer commitment*
***H***_**4**_ *: Customer experience has an impact on corporate image*

### Mediating Role of Corporate Image

As researchers, we are doing our best to identify the elements that have a significant impact on consumer purchasing decisions in the financial sector. Several studies have shown that corporate image has a substantial impact on customer behavior and business efficiency. Consumers, investors, and various stakeholders all observe the company's brand or image, which establishes an image and reputation. As a result, businesses are now becoming increasingly conscious of the importance of preserving and enhancing their corporate image among the stakeholders. To protect the company's reputation in a competitive market, the corporate image must represent the company's goals, beliefs, and ethics. It aids the organization in distinguishing its image from that of its rivals by presenting a sense of originality (Zameer et al., 2015).

The corporation's goals and ideals should be expressed in both forms, that is, visual and non-visual. Trademarks such as emblems, monograms, advertisements, and clothing make up the visual aspect. Training techniques, techniques, and language were all included in the non-visual format. Corporate image is malleable and could be influenced by external circumstances. The administration shall continually consider the organization's image and reputation. Administrators have acknowledged the value of the corporate image in recent years, but it is tough to explain it to various audiences (Herstein et al., 2008). The spectrum of interpretations that are retained in the brains of stakeholders is represented by the corporate image. It is primarily formed by technical skill, such as how the audience perceives the quality service, and it is also generated by functional value, which includes how products are provided (Zameer et al., 2015).

Based on the literature discussed above in previous sections, service quality had shown its impact on the corporate image (Chenet et al., 2010; Özkan et al., 2020). It has also been studied and investigated as a mediator in different contexts having a facilitating role in organizational management regarding customers' purchase activities. Some of the investigations focused on the mediating role of the corporate image between service quality and customer satisfaction (Chien and Chi, 2019). Some of the researchers also found the significant mediating role of the corporate image between service quality and students' loyalty (Yingfei et al., 2021). This literature support proposed that there could have been a mediating role of the corporate image between service quality, customer experience, and customer commitment as well. Therefore, the following hypotheses were suggested.

***H***_**5**_ *: There could be a possible mediation of corporate image between SQ and customer commitment*
***H***_**6**_ *: There could be a possible mediation of corporate image between CE and customer commitment*

**Figure 1:**
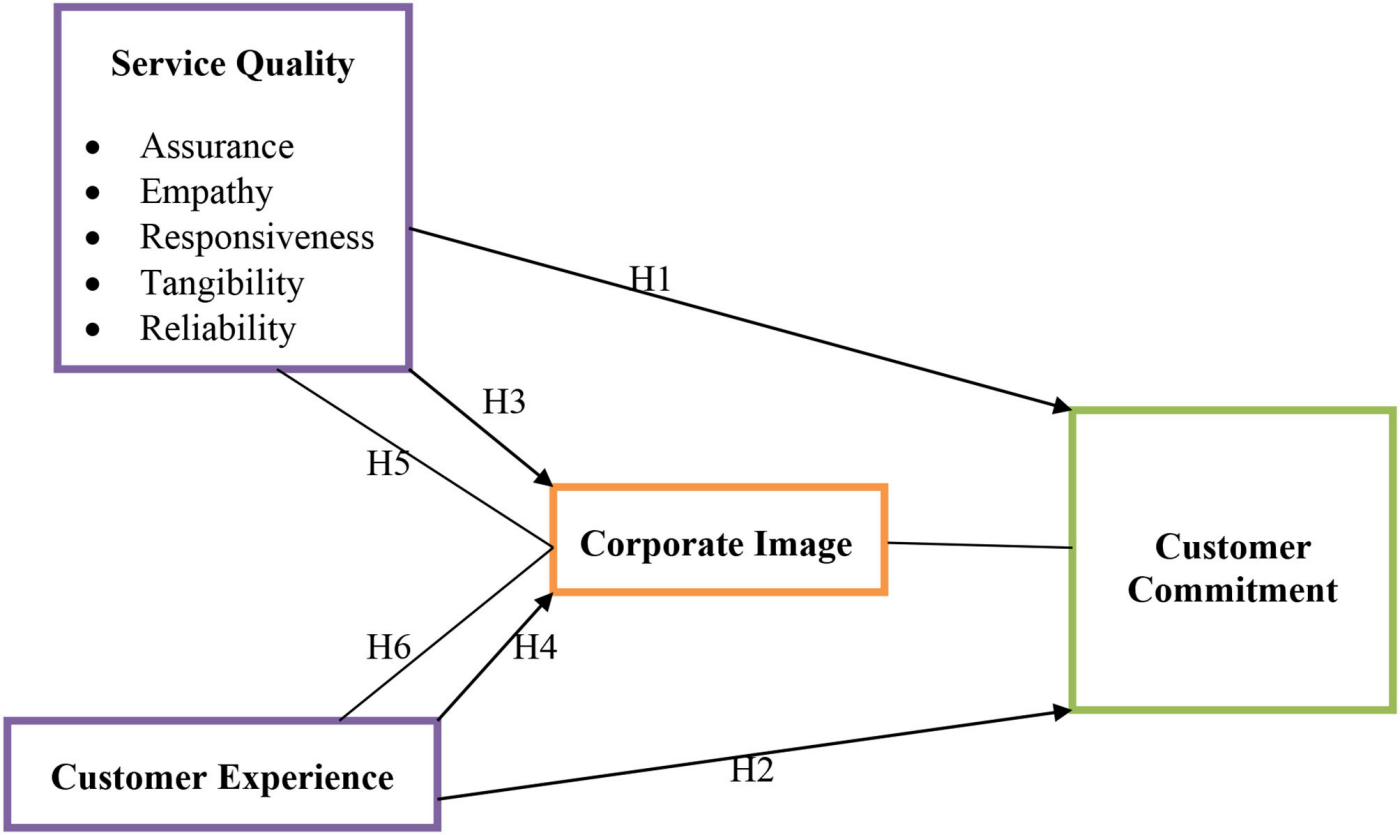
Theoretical model.

*Based on the above literature support and hypothesis following framework has been developed (see* *Figure 1**)*.

## Methodology

**Figure 2:**
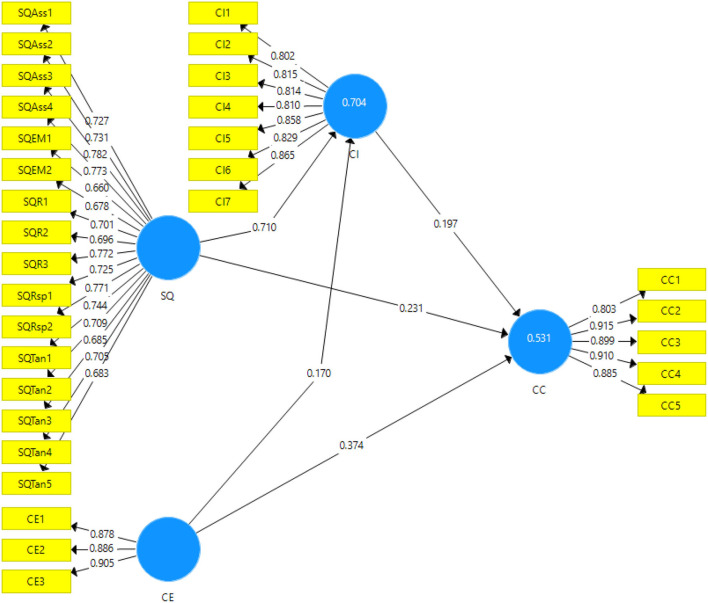
Output of measurement model algorithm. SQ, Service quality; CE, Customer experience; CI, Corporate image; CC, Customer commitment.

**Figure 3:**
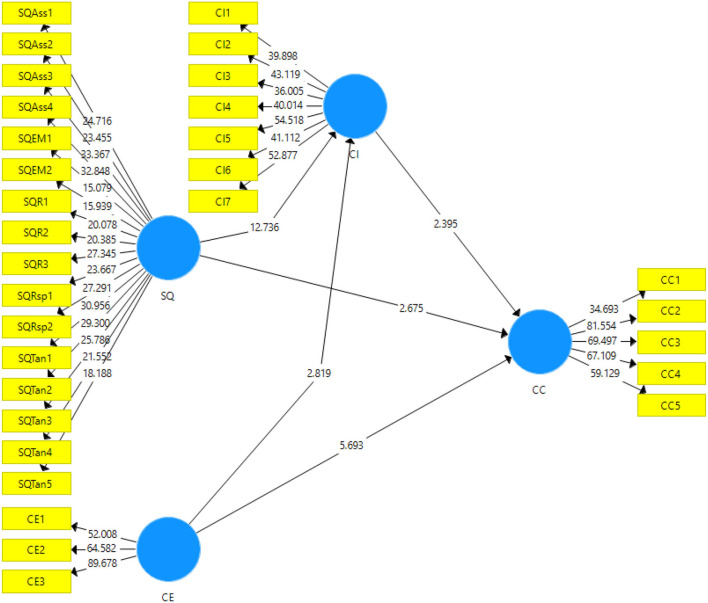
Output of structural model Bootstrapping. SQ, Service quality; CE, Customer experience; CI, Corporate imagel; CC, Customer commitment.

For analysis purposes, the quantitative research design has been chosen with the deductive approach. It helps to confirm/deject the hypotheses based on the relationships quantified, found significant or not. Certain tests are proposed in the research to reach the conclusion. This approach helps in reducing the chances of any bias in the statistical analysis. In this study, the data collection was conducted through self-administered surveys to acquire data from the potential participants. The population frame used in this study is the logistic organizations that are offering logistic services in numerous supply chains. The convenience sampling technique has been used in this study to choose the sample (Etikan et al., 2015). It is considered beneficial because of the convenient availability of the participants along with cost-effective and time-saving abilities (Avotra et al., 2021; Nawaz et al., 2022). The collected data was run for statistical analysis to reach the conclusions regarding the hypotheses formulated in the literature review. The sample size in this study was 366. The unit of analysis in this study was the customers of the organizations that provide logistic services (Centobelli et al., 2020). The time horizon was cross-sectional as the data were collected at one point in time. The ethical considerations of the research had been met by letting the respondents fill out the survey anonymously and it was promised to maintain their anonymity. The data were analyzed using the software Smart-PLS version 3.3.5, as it is a vigorous and robust software that easily deals with the non-normal and small data sizes giving accurate results. This uses partial least square structural equation modeling to analyze. Furthermore, it analyzes the data in two stages by outputting two models: the inner model (structural model) and the outer model (measurement model) (see Figures 2, 3).

### Measurement

The study has used the questionnaire survey method for the purpose of data collection. The questionnaire had been made on a 5-point Likert scale (1–5) where 1 represented “strongly disagree” and 5 represented “strongly agree.” The scale for the variable of service quality addressing the five facets of empathy (2-items), assurance (4-items), responsiveness (2-items), reliability (3-items), and tangibility (5-items) have been addressed in the composite variable of service quality consisting of 16 items in total taken from the study by (Chien and Chi, 2019). The scale for the variable of a corporate image consisting of seven items was also taken from the study by (Chien and Chi, 2019). The scale for the variable of customer experience consisting of three items was taken from the study by (Bawack et al., 2021). The scale for the variable of customer commitment consisting of five items was taken from the study by (Yilmaz Uz, 2019). All these scales had been screened in the initial stage using validity and reliability tests.

### Demographics Details

The results for the demography of the respondents show that male and female participation in the study was almost equal. While, the highest number of participants showed they possessed a bachelor's degree followed by master's. The highest participation had been seen from the age group of 31 to 40 years followed by 41 to 50 years. Further, most of the respondents had an experience of <1 year with their service provider followed by 1–3 years and so on. The results of the demographic analysis can be seen in Table 1.

**Table 1:** Demographics analysis.

**Demographics**	**Frequency**	**Percentage**
**Gender**		
Male	188	51.36%
Female	178	48.63%
**Age (years)**		
20–30	57	15.57%
31–40	119	32.51%
41–50	105	28.68%
Above 50	85	23.22%
**Education**		
Bachelors	167	45.62%
Masters	120	32.78%
Ph.D. and others	54	14.75%
**For how long you are using this**		
**particular firm's services? (years)**		
<1	115	31.42%
1–3	96	26.22%
4–6	75	20.49%
>6	80	21.85%

*N = 366*.

## Data Analysis and Results

The analytical technique used for data analysis in the study is structural equation modeling. The details for the data analysis are given below.

### Measurement Model

The figure obtained for the measurement model is given below (see Figure 2).

Table 2 shows the output statistics of the measurement model. It includes the convergent validity which consists of factor loadings, average variance extracted, and the variance inflation factors. The cut-off margin given in the literature for the factor loadings is 0.6 (Xiaolong et al., 2021). In present study, all the items have been loaded with factor loadings on their respective factors with more than 0.6. In the present study, all the values have been reported above 0.6, thus indicating significant factor loading of the items on their respective variables. The average variance extracted, according to Archer et al. (2021), should be above 0.5. In the present study, Table 2 shows that all the values for AVE are above 0.5 indicating a substantial explanation of the variance by the items of the variables. Similarly, the last measure for the convergent validity is the variance inflation factor. The value of VIF, according to (Craney and Surles, 2007; Waheed and Baig, 2017), should be <5.5 to show that the issue of collinearity is not established among the variables or items. In the present study, all the values for VIF meet the acceptance range as given in the literature.

**Table 2:** Outer model (measurement model).

**Variables**	**Factor loadings**		**Cronbach alpha**	**Composite reliability**	**AVE**	**VIF**
**Customer commitment**	CC1	0.803				1.946
	CC2	0.915				4.001
	CC3	0.899	0.929	0.947	0.780	4.198
	CC4	0.910				4.885
	CC5	0.885				3.051
**Customer experience**	CE1	0.878				2.142
	CE2	0.886	0.868	0.919	0.791	2.282
	CE3	0.905				2.450
**Corporate image**	CI1	0.802				3.378
	CI2	0.815	0.923	0.938	0.685	3.519
	CI3	0.814				2.359
	CI4	0.810				2.528
	CI5	0.858				3.408
	CI6	0.829				3.009
	CI7	0.865				3.332
**Service quality**	SQAss1	0.727				4.311
	SQAss2	0.731	0.939	0.946	0.522	3.457
	SQAss3	0.782				2.635
	SQAss4	0.773				2.865
	SQEM1	0.660				2.373
	SQEM2	0.678				2.879
	SQR1	0.701				3.569
	SQR2	0.696				3.679
	SQR3	0.772				4.936
	SQRsp1	0.725				4.373
	SQRsp2	0.771				5.066
	SQTan1	0.744				2.818
	SQTan2	0.709				3.535
	SQTan3	0.685				2.947
	SQTan4	0.705				1.932
	SQTan5	0.683				3.086

The reliability of the scales explains the internal consistency of the scales used for a particular study. The scales used in the present study have been checked through the Cronbach alpha and the composite reliability. According to Bujang et al. (2018), the values for Cronbach alpha and the composite reliability should be above 0.7. In the present study, all variables of the study show significant statistics for these criteria, thus falling within the acceptance range.

The distinction among the variables measuring the related concepts has been checked with the help of discriminant validity. For this, the tests of heterotrait monotrait ratio (HTMT) and the Fornell and Larcker criteria have been used. These two tests are the most commonly used tests for checking the discriminant validity of the scale (Nawaz et al., 2019, 2021). Table 3 shows the Fornell and Larcker Criteria results. This table indicates that all the values obtained in this table have the highest values at the top of each column indicating the absence of collinearity and a higher degree of distinction indicating the presence of discriminant validity (Fornell and Larcker, 1981). Similarly, the second measuring test is the HTMT ratio, which also helps in indicating the presence of a higher degree of correlation or collinearity among the variables. Table 4 indicates the results of the test. According to Franke and Sarstedt (2019), the values obtained in the table should be <0.9 for scales to pass the criteria of discriminant validity. In this study, all the values obtained through the HTMT ratio are all below 0.9 indicating the presence of discriminant validity.

**Table 3:** Fornell and larcker criteria.

	**CC**	**CE**	**CI**	**SQ**
CC	0.883			
CE	0.672	0.890		
CI	0.642	0.676	0.828	
SQ	0.661	0.713	0.831	0.722

*SQ, Service quality; CE, Customer experience; CI, Corporate image; CC, Customer commitment*.

**Table 4:** HTMT ratio.

	**CC**	**CE**	**CI**	**SQ**
CC				
CE	0.746			
CI	0.684	0.746		
SQ	0.688	0.773	0.882	

*SQ, Service quality; CE, Customer experience; CI, Corporate image; CC, Customer commitment*.

R-square values obtained in this study have been found really good. R-square indicates the model fit through regression fit, also known as the coefficient of determination. In this study, the highest r-square value has been obtained for the variable corporate image which is 70%. It is followed by the other variable customer commitment which shows the r-square value of 52.7%. The f-square value shows the effect size among the relationships established in the literature. In this study, the highest f-square value has been obtained for the relationship between service quality and corporate image which is 0.83 which is a large effect size. It is followed by the relationship between customer experience and customer commitment which is 0.14 and it is considered as a medium effect size.

### Structural Model

The structural model, also known as the inner model, has been used to accept or deject the hypotheses of the study. For this purpose, the study used the structural equation modeling technique considering the t-statistic and the *p*-values. It is done through the resampling technique of bootstrapping at a 95% confidence interval. Therefore, the hypotheses are accepted or rejected at 5% (Andrade, 2019). Output for the structural model is given in Figure 3.

Results of the study showing direct effects have been given in Table 5. The findings have indicated that service quality has a significant and positive impact on the customer commitment toward the logistic services. This indicates the acceptance of the first hypothesis that service quality has an impact on customer commitment with t-statistic = 2.67, *p* < 0.05 (H1). The results supported the second hypothesis of the study that postulated that customer experience has an impact on customer commitment with t-statistic = 5.69, *p* < 0.05. The results for the third hypothesis showed t-statistic = 12.73, *p* < 0.05; therefore, it is accepted that service quality has an impact on the corporate image. The fourth direct effect of the study was also accepted with t-statistic = 2.81, *p* < 0.05; therefore, it is accepted that customer experience has a positive impact on the corporate image of the organizations.

**Table 5:** The direct effects of the variable.

**Paths**	**H**	**O**	**M**	**SD**	**T-Statistic**	***P*-value**	**Results**
SQ -> CC	H_1_	0.231	0.234	0.086	2.675	0.008	*Accepted*
CE -> CC	H_2_	0.374	0.374	0.066	5.693	0.000	*Accepted*
SQ -> CI	H_3_	0.710	0.713	0.056	12.736	0.000	*Accepted*
CE -> CI	H_4_	0.170	0.169	0.060	2.819	0.005	*Accepted*

The indirect effects of the study have been reported in Table 6. The first indirect hypothesis is about the mediating role of the corporate image between the relationship of service quality and customer commitment. The results of the data analysis show t-statistic = 2.46, *p* < 0.05, thus supporting the hypotheses and accepting H5. Furthermore, the second indirect hypothesis is about the mediating role of the corporate image in the relationship between customer experience and customer commitment. The results of the hypotheses show t-statistic = 1.54 and *p* > 0.05 indicating that data do not support the hypotheses, hence rejected (H6).

**Table 6:** Indirect effects.

**Paths**	**H**	**O**	**M**	**SD**	**T-Statistic**	***P-*value**	**Results**
SQ -> CI -> CC	H_5_	0.034	0.034	0.022	1.541	0.124	Rejected
SQ -> CI -> CC	H_6_	0.140	0.136	0.057	2.467	0.014	*Accepted*

## Discussion

This study was conducted to evaluate the impacts and roles of service quality and customer experience on customer commitment. This research was focused on finding the associations between these aspects of customers and producers of the services and products. This empirical study also tried to find out the mediating role of the corporate image between service quality, customer experience, and customer commitment. This kind of relationship of the corporate image is necessary as it provides an indication of CSR at the firm level. The customers are influenced by the corporate image of the firm. It happens because the corporate image has been used as a tool for implementing CSR at the organizational level. The association of service quality, customer experience, and corporate image was also analyzed in this study. Previously, research has been done in the direction of associations between service quality, corporate image, and customer satisfaction (Chien and Chi, 2019). Similarly, some of the researchers tried to find out the relationship between customer experience and customer commitment and found significant and positive associations (Keiningham et al., 2017).

This study found a significant and positive association between service quality and customer commitment. These kinds of results are supported by the results of (Chien and Chi, 2019), as service quality affected the behaviors of the customers positively in that research. Customer satisfaction has been affected by service quality in their research which leads to customer commitment. Hence, it is assumed that customer commitment to the services and products of the brands is the depiction of their satisfaction with the quality of services. This research assessed the commitment of customers regarding logistics and delivery of products and services. Similar results have also been obtained previously in the investigations evaluating the impact of service quality on customer satisfaction which leads to customer commitment in the context of logistics and delivery of digital purchases (Buldeo Rai et al., 2019). This study also looked into the relationship of service quality with corporate image, which also showed a positive and significant association. It indicated that if the quality of services is improved in terms of empathy, assurance, tangibility, responsiveness, and reliability, then it could lead to developing a better corporate image. These dimensions were also tested by (Uzir et al., 2021) in evaluating the impact of service quality on corporate image and customer satisfaction. The results were similar to current research which supported the association between service quality and corporate image. Other direct relationships of customer experience, corporate image, and customer commitment were also studied in this research. The results indicated that customer experience had a strong association with both the corporate image and customer commitment.

These results might be obtained because the experience of customers plays an important role in their decision-making about buying and not buying the products and services of the companies. These results are also supported by the fact that customer experiences are multidimensional constructs concentrating on their cognitive, emotional, behavioral, sensory, and social responses to a firm's products across the complete buying experience (Lemon and Verhoef, 2016). Therefore, a sense of satisfaction arises among the customers which leads to a commitment to products and services. These results are also in agreement with a few scholars in the recent past who tried to find out the relationship between customer experience and customer commitment and found significant and positive associations (Keiningham et al., 2017). The indirect or mediating effects of the corporate image were also tested in this study. The results indicated that there was not a positive mediation of corporate image between service quality and customer commitment. These contradictory results to previous research by (Uzir et al., 2021) indicated that there was no need to spend more on developing a corporate image when the organization was already providing good quality services to the customers. It could not aid in boosting the relationship of service quality with customer commitment. The possible reason may lie in the fact that if brands provide assurance, reliable product, tangibility, empathy, and proper responses, then customers do not need to go through the established image of the products and services of the corporation.

The last indirect effects of the corporate image between customer experience and customer commitment were significant, indicating that only customer experiences were not enough for developing a sense of commitment in the customers for buying activities of products and services of brands. It aided in the building of customer commitment. Previously, it also has been explored and investigated as a mediator in a variety of scenarios, with a facilitating role in organizational management when it comes to customer purchasing activities. Some of the studies looked at the impact of company image in mediating the relationship between service quality and customer happiness (Chien and Chi, 2019). Some investigations discovered that company image plays an important role in mediating the relationship between SQ and student loyalty research (Hassan et al., 2020).

### Managerial Implications

The findings of this study imply certain implications as well for the service quality of the logistic industry. First of all, it is important for the organizations to improve the service quality so they would enrich their corporate image among the customers which would help them access and penetrate new markets. The word of mouth has always been a major source of informal marketing that brings the business to the firms (Fazli-Salehi et al., 2019). Further, it is also important that the logistic industry finds new ways of collaboration to strengthen their services to pick a major chunk in the market that contributes to achieving a better corporate image and ultimately a better satisfaction level of the customers.

### Theoretical Contribution

The present study has contributed to the literature on the service industry and service quality by examining how and to what extent customer experience and service quality plays a role in customer commitment. It further has added to the literature that service quality and customer experience have a positive and significant impact on the corporate image of the service providers. This also has shown that the corporate image of the service providers is an important and significant mediating factor in bridging the effect of service quality in the customer commitment.

### Limitations and Future Research

Like other research studies, this study also indicates certain limitations. First of all, the results of the present study have been drawn based on only one service industry that is logistics which may show some biases in the results. Therefore, it is recommended that this study is conducted in other service-providing industries as well to endorse the findings of the present study. Second, the present study has measured the mediating role of corporate image between service quality and customer commitment, as it seems interesting to conduct this study with a moderating variable to see if the leadership of the organizations play role in affecting the customer commitment. Furthermore, it is recommended to study other mediating variables such as technology, digitalization on the customer retention that they have modified the approaches and perspectives of the customers in the services industry.

## Conclusion

Investigating the important drivers of corporate image toward customer commitment has been crucial in this competitive scenario logistic industry especially after the post-pandemic situation, since customer experience and service quality have been considered the important factors in this situation. Therefore, this study has attempted to investigate the role of service quality (considering the five major components of service quality: responsiveness, empathy, reliability, tangibility, and assurance) and customer experience in maintaining the corporate image of logistic service providing firms and how it consequently affects the customer commitment. This study was carried out with the help of the customers of service providing logistic industry in China. The study has found that service quality and customer experience play a vital role in building the corporate image which further contributes to the customer commitment to these organizations. Moreover, the study has also found a significant mediating role of the corporate image in the relationship between service quality and customer commitment.

## Data Availability Statement

The original contributions presented in the study are included in the article/supplementary material, further inquiries can be directed to the corresponding author.

## Author Contributions

ZM conceived and designed the concept. BK-H collected the data and supervised. YY wrote the article. All authors read and agreed to the published version of the manuscript.

## Funding

The study was funded by scientific project code (21BJY056) Research on the Mechanism and Path of High-quality Development of the Innovation-Driven Trade under the New Pattern of Double Cycle. The authors would like to give their appreciation for the acknowledgment of the Ningbo Key Research Base for Philosophy and Social Studies (Regional Open Cooperation and Free Trade Zone Research Base) for the valuable contribution to the success of our project.

## Conflict of Interest

The authors declare that the research was conducted in the absence of any commercial or financial relationships that could be construed as a potential conflict of interest.

## Publisher's Note

All claims expressed in this article are solely those of the authors and do not necessarily represent those of their affiliated organizations, or those of the publisher, the editors and the reviewers. Any product that may be evaluated in this article, or claim that may be made by its manufacturer, is not guaranteed or endorsed by the publisher.
